# Behind the Scenes: A Pilot Study on the Evaluation of Healthcare Professionals’ Approaches to Transitional Care in Adolescents With Chronic Gastrointestinal Disorders

**DOI:** 10.7759/cureus.68092

**Published:** 2024-08-29

**Authors:** Silvia Cristina Poamaneagra, Elena Tataranu, Gabriela Stefanescu, Cristiana Mihaela Andronic, Gheorghe G Balan, Georgiana Emmanuela Gilca-Blanariu, Ileana Ioniuc, Catalina Mihai, Liliana Anchidin-Norocel, Smaranda Diaconescu

**Affiliations:** 1 Pediatrics, George Emil Palade University of Medicine, Pharmacy, Science, and Technology, Targu Mures, ROU; 2 Pediatrics, Faculty of Medicine and Biological Sciences, Stefan cel Mare University of Suceava, Suceava, ROU; 3 Gastroenterology, Faculty of Medicine, Grigore T. Popa University of Medicine and Pharmacy, Iasi, ROU; 4 Pediatrics, Faculty of Medicine, Grigore T. Popa University of Medicine and Pharmacy, Iasi, ROU; 5 Nutrition, Faculty of Medicine and Biological Sciences, Stefan cel Mare University of Suceava, Suceava, ROU; 6 Pediatrics, Faculty of Medicine, Titu Maiorescu University of Medicine, Bucharest, ROU

**Keywords:** transfer age, transition age, transition barriers, transition needs, healthcare specialists, transition

## Abstract

Background: Due to recent advances in healthcare, more and more teenagers with chronic diseases emerge into adulthood, posing challenges for both pediatric and adult healthcare systems. The transition from pediatric to adult healthcare settings presents a complex and pivotal phase for adolescents managing chronic conditions. This process necessitates collaboration among adolescents, parents, pediatric specialists, and adult healthcare providers.

Aims: This study aims to assess the current practices of doctors caring for teenagers with chronic digestive diseases and to identify the needs and barriers experienced by professionals during the transition of their patients.

Methods: In order to achieve the aims of this study, we employed a cross-sectional survey study. Pediatric gastroenterologists (PG), general pediatricians (GP), adult gastroenterologists (AG), and primary care physicians (PCP) applied a 20-closed multiple-choice questionnaire.

Results: There were 70 responders; 90% did not follow a transition program. The ideal age for beginning the transition was considered 16-17 (51.4%), and the transfer was recommended at 19-20 years of age by 42.9% and at 18 by 45.7%. Regarding the resources, 78.55% required an online platform, 58.55% solicited transition readiness assessments, 51.41% identified online forms distributed through social media, 48.55% selected brochures for patients and families, and a guideline for medical practitioners. Both GPs and PGs (0.836, p<0.05) requested higher numbers of resources. Identified barriers were the absence of a transition expert (65.7%), lack of time for individualized transition programs (61.41%), patients’ psycho-emotional attachment to the pediatric team (52.84%), and adolescents' lack of disease knowledge (57.13%).

Conclusions: Investigating the roles and challenges faced by healthcare professionals during the transitional period is crucial for optimizing continuity of care, enhancing patient outcomes, and addressing systemic gaps in healthcare delivery. We identified valuable tools that could be used in transition programs applicable to all institutions caring for adolescents with chronic diseases.

## Introduction

Recent advances have been made regarding the diagnostic, treatment options, and accessibility for pediatric patients with chronic diseases. Due to the perpetual research with more and more promising results, a survival improvement has been noticed, and a significant number of teenagers reach adulthood and are candidates for transition from the family-centered healthcare system towards the adult-oriented system.

International forums, such as the European Society of Pediatric Gastroenterology, Hepatology, and Nutrition and the European Association for the Study of the Liver in 2018, the North American Society for Pediatric Gastroenterology, Hepatology, and Nutrition in 2016, the European Crohn’s and Colitis Organization in 2017 and 2020, and the Crohn’s and Colitis Foundation in 2022, have already issued guidelines for the transition of various chronic gastrointestinal diseases [1-6].

Despite the rising interest in international transition protocols, in Romania, there is no standard national transition protocol and no reported data on any isolated or sporadic practices. 

In order to establish an effective strategy for a successful national transition protocol, one must take into account the meeting of the needs of families, patients, but also of specialists, and healthcare providers.

The aims of this study were to assess medical practices used in the process of transition of pediatric patients suffering from chronic digestive diseases and to identify and report the needs, resources, and barriers experienced by specialists caring for such children.

## Materials and methods

In order to achieve the aims of this study, we employed a cross-sectional study using the survey methodology.

Regarding the inclusion criteria, we included specialists in any of the following specialties: general pediatrics (GP), pediatric gastroenterology (PG), adult gastroenterology (AG), and primary care physicians (PCP). We excluded resident physicians or doctors with multiple specialties currently working outside the previously mentioned specialties from participating in the study.

We contacted 98 doctors who expressed willingness to participate in the study and provided their phone numbers at medical conferences. They were subsequently asked to anonymously share their experiences regarding current transition practices, transition policy awareness, barriers, and needs they may have encountered during this process through an online questionnaire.

The questionnaire consisted of 20 questions and was created by our research team. The questionnaire was evaluated by two teams formed by one pediatric gastroenterology specialist and two gastroenterology and hepatology specialists, and their notes and comments on the questionnaire were analyzed by a third team formed by one professor of pediatric gastroenterology and one professor of gastroenterology and hepatology.

During the next step of the validation process, two experts in the field (one professor of pediatric gastroenterology and one professor of gastroenterology and hepatology) completed a tabulation form where they appreciated the relevance and clarity of questions. Based on their items, we calculated the item-content validity index. The score-content validity index average was 0.93, the proportion relevance for Expert 1 was 0.90, and for Expert 2 it was 0.95. Based on these findings, the score-content validity index (universal agreement) average was 0.85. These results allowed us to use the questionnaire with a satisfactory level of content validity.

The questionnaire was distributed online using Google Forms (Google Inc., Mountain View, CA), via WhatsApp (Meta Platforms, Inc., Menlo Park, CA), and by email with an invitation to participate in the study to doctors from the database of the Romanian Association of Pediatric Education of Family Doctors. We employed a purposive sampling technique, allowing us to target specific subgroups of interest within the studied population.

By accessing the link, the participants received information about the study, details on personal data confidentiality, and the participation agreement, in compliance with the existing regulations on data protection. The questionnaire was available for 10 days, from 04.03.2024 to 14.03.2024. 

The study was approved by the research and ethics committee of St. Mary Hospital, Iasi, Romania (decision no. 38761/09.11.2020).

To investigate the collected data, we used IBM SPSS Statistics software for Windows, version 29.0.2.0 (IBM Corp., Armonk, NY), for the statistical analysis, the XLSTAT 2024 (Lumivero, Denver, CO), and the Minitab 22.1 (Minitab, LLC., State College, PA) for illustrating the results as a correlogram. Quantitative variables have been expressed as numbers and/or percentages. All statistical tests are two-tailed.

## Results

In this pilot study, a total of 98 doctors were contacted via online forms, and 70 doctors completed the online survey (71.42%). Of these, 21 (30%) were PGs, 20 (28.6%) were GPs, 13 (18.6%) were PCPs, and 16 (22.8%) were AGs. Because of the heterogenicity of the studied population, participants were asked not to answer questions they felt did not apply to their medical specialty. The majority of respondents worked in urban areas (n = 68; 97.1%).

Transition protocols for adolescents suffering from chronic diseases were found to be almost nonexistent, with only a small portion of responders benefiting from such protocols: seven (10%) versus 63 (90%). The need for a multidisciplinary team to coordinate the process was identified by 68 (97.1%) participants. Transition practices were assessed, and the results are available in Table 1.

**Table 1:** Distribution of transition practices and participants’ recommendations regarding the optimal age for beginning the transition process and for the medical transfer

Transition practices		Number of responders	Percentage (%)
By issuing the last medical letter		34	50%
Through discussions held strictly with the parents during the last medical visits		6	8.8%
Through discussions with both parents and patients during the last visits		26	38.2%
Through discussions in which an adult specialist also participates		1	1.5%
Following a clinic/hospital/office protocol		1	1.5%
Recommendations regarding beginning the transition process and the medical transfer			
The ideal age for beginning the transition process	12-14 years old	4	5.7%
	15-16 years old	16	22.9%
	16-17 years old	36	51.4%
	17-18 years old	14	20 %
The ideal age for a transfer	17 years old	7	10%
	18 years old	32	45.7%
	19-20 years old	30	42.9%
	21 years old	1	1.4%

In the context of assessing the transition readiness, the independence and knowledge levels of adolescents were evaluated; 51 (73.9%) respondents believed their patients were not ready to independently make health-related decisions. While 46 (65.7%) participants reported encouraging patients to self-manage their chronic conditions, only 39 (55.7%) promoted independent consultations for teenagers.

Regarding patient knowledge of their chronic disease, medication, potential side effects, and complications of untreated conditions, 45 (65.2%) specialists claimed their patients had some information but lacked specificity or details. Additionally, 19 (27.5%) participants believed their patients were not adequately informed, and only five (7.2%) expressed confidence in their patients' understanding of their disease. 

Nineteen (31.7%) responders had been contacted by patients or their families for advice or care after the age of 18 for medical emergencies, 20 (33.3%) for disease management advice, and 18 (30%) for adult-oriented center referrals.

The importance of adolescents independently scheduling consultations or renewing prescriptions was rated on a Likert scale from one (not important) to five (very important), and results are available in Table 2.

**Table 2:** Assessment of the importance of adolescents independently scheduling consultations or renewing prescriptions

Importance of adolescents independently scheduling consultations or renewing prescriptions	1 (not important)		2		3		4		5 (very important)	
	n	%	n	%	n	%	n	%	n	%
	0	0	2	2.9%	1	1.4%	22	31.4%	45	64.3%

Particular behaviors in young adults have been identified, such as reluctance regarding the treatment and investigations (five), hesitation to come to consultations without their parents, low compliance to treatment (two), as well as difficulties in communication (three).

**Figure 1:**
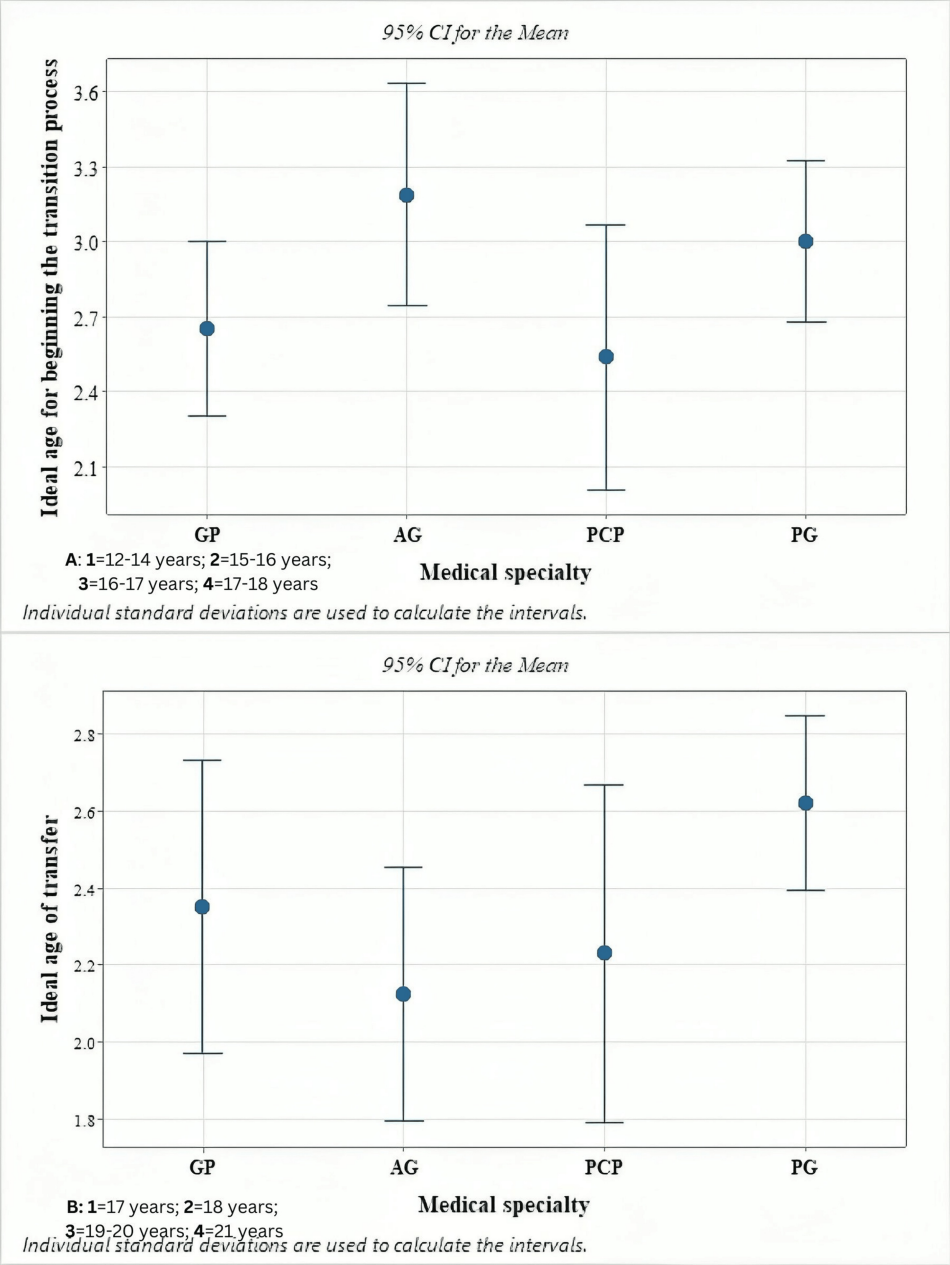
Recommendations on the ideal age of beginning the transition process and for the medical transfer The y-axis (Figure 1A), represented numerically from 1 to 4, was chosen to simplify data evaluation and interpretation. This approach was taken to provide a clear and concise representation of medical preferences regarding the ideal age to start the transition process, replacing the age ranges with values as follows: 12-14 years: 1; 15-16 years: 2; 16-17 years: 3; 17-18 years: 4). B. To facilitate the analysis and interpretation of the data, the values on the y-axis are represented numerically from 1 to 4, replacing the age intervals with values such as 17 years: 1, 18 years: 2, 19-20 years: 3, 21 years: 4. This method may facilitate the understanding and application of study results by providing a more user-friendly framework for managing the transition process of pediatric patients with chronic medical conditions. GP: general pediatricians; AG: adult gastroenterologists; PCP: primary care physicians; PG: pediatric gastroenterologists

The ideal age for the onset of a transition process was considered to be between 16 and 17 years of age by 36 (51.4%) responders; 16 (22.9%) considered this process should start at 15-16 years of age (Table 1). Recommendations on beginning the transition by various specialists are illustrated in Figure 1.

Regarding the ideal age for the medical transfer, 30 (42.9%) participants stated it should happen between the ages of 19-20, while 32 (45.7%) felt it should take place at 18 years of age (Table 1).

Pearson correlations carried out to identify a possible association between medical specialties recommending transfer at older ages and contact by patients or their families either for emergencies or for medical advice with regard to chronic disease management are synthesized in Table 3.

**Table 3:** A. Pearson correlation between later transfer recommendations and doctors being contacted by patients or families for emergencies or advice for disease management after the age of 18; B. Pearson correlation between medical specialties and the need for a high number of resources; C. Pearson correlation between medical specialties and identified barriers. Values marked with * are different from 0 with a significance level of alpha = 0.05. PG: pediatric gastroenterologists; GP: general pediatricians; AG: adult gastroenterologists; PCP: primary care physicians

	R values					p<0.05
	Variables	Under 18 years	Over 18 years	Contacted		
A. Correlations between later transfer recommendations and doctors being contacted by patients or families for emergencies or advice after the age of 18	Under 18 years	1*				
	Over 18 years	-0.223	1*			
	Contacted	-0.171	0.617*	1*		
Variables		GP	AG	PCP	PG	
B. Correlations between medical specialties and the need for a high number of resources	GP	1*				
	AG	0.489	1*			
	PCP	0.414	-0.393	1*		
	PG	0.836*	0.686*	0.079	1*	
C. Correlations between medical specialties and identified barriers	GP	1*				
	AG	0.959*	1*			
	PCP	0.751*	0.710*	1*		
	PG	0.612*	0.455	0.549*	1*	

Subsequently, we sought to identify doctors’ needs and barriers influencing their practice. Regarding resources, 34 (48.55%) participants preferred brochures for patients and families, 35 (49.98%) requested systematic guidelines for doctors and nurses, and 41 (58.55%) identified a need for a transition readiness assessment questionnaire. Additionally, 36 (51.41%) responders wanted online forms for social media and online communication applications, 30 (42.83%) found an online patient's medical history useful, and 55 (78.55%) desired an online platform with comprehensive information accessible to patients during the transition process (Table 4).

**Table 4:** A. distribution of resource preferences depending on medical specialties; B. distribution of barriers identified by each medical specialty PG: pediatric gastroenterologists; GP: general pediatricians; AG: adult gastroenterologists; PCP: primary care physicians

A. Resources	GP	AG	PCP	PG
Brochures for patients and their families	12 (17.14%)	5 (7.14%)	8 (11.42%)	9 (12.85%)
A systematic guideline for doctors and nurses	9 (12.85%)	7 (10.00%)	8 (11.42%)	11 (15.71%)
Group educational sessions	5 (7.14%)	4 (5.71%)	5 (7.14%)	4 (5.71%)
Transition readiness assessment questionnaire (including information regarding the chronic disease)	11 (15.71%)	9 (12.85%)	3 (4.28%)	18 (25.71)
Online forms that could be distributed through social media or online communication devices	10 (14.28%)	6 (8.57%)	9 (12.85%)	11 (15.71%)
Online patient's medical history	8 (11.42%)	11 (15.71%)	2 (2.85%)	9 (12.85%)
An online platform containing all the information that could be accessed by patients in case they encounter problems during the transition process.	16 (22.85%)	11 (15.71%)	8 (11.42%)	20 (28.57%)
B. Barriers				
Lack of specialized centers dedicated to chronic patients during the peri-transfer period	8 (11.42%)	7 (10.00%)	7 (10.00%)	13 (18.57%)
Psycho-emotional attachment of the patient and their family to the pediatric team	11 (15.71%)	11 (15.71%)	7 (10.00%)	8 (11.42%)
The absence of a transition expert for each center	11 (15.71%)	11 (15.71%)	11 (15.71%)	13 (18.57%)
Adolescents’ inability to take care of their own health as a main barrier	4 (5.71%)	5 (7.14%)	3 (4.28%)	4 (5.71%)
Lack of time to create an individualized transition program	12 (17.14%)	12 (17.14%)	8 (11.42%)	11 (15.71%)
Adolescents' lack of knowledge regarding chronic disease, the followed medication and the possible evolution of the disease in the absence of medication	10 (14.28%)	10 (14.28%)	6 (8.57%)	14 (20.00%)
Shortage of doctors in the primary care network to assist in the transition process	9 (12.85%)	8 (11.42%)	7 (10.00%)	10 (14.28%)

**Figure 2:**
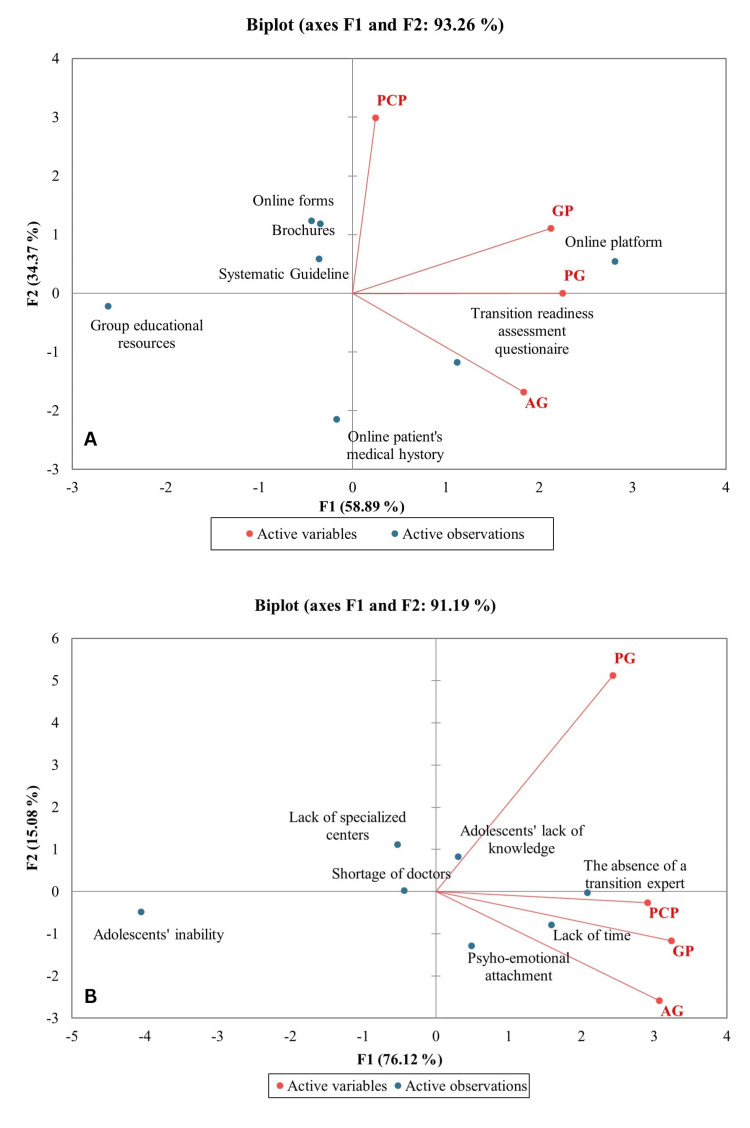
The PCA of select resources and identified barriers GP: general pediatricians; AG: adult gastroenterologists; PCP: primary care physicians; PG: pediatric gastroenterologist; PCA: Principal Component Analysis

The Principal Component Analysis (PCA) method limited the data into two principal components covering 93.26% of the variability (F1: 58.89% and F2: 34.37%). The analyzed resources, such as brochures for the patients and families, a systematic guide for doctors and nurses, and online forms distributed through social media or online communication applications, were grouped around PCP. The transition readiness assessment questionnaire and an online platform are distributed in this PCA as being specific to PG and GP. As for AG, they usually consider the online patient’s medical history necessary (Figure 2).

Pearson correlation carried out in order to identify possible associations between the medical specialty and the need for a high number of resources has been synthesized in Table 3.

Specialists identified several barriers to effective transition: 34 (48.55%) cited a shortage of PCP, 35 (50%) noted the lack of specialized centers for chronic patients during the peri-transfer period, and 37 (52.84%) mentioned the psycho-emotional attachment of patients and families to the pediatric team; 40 (57.13%) indicated adolescents' lack of knowledge about their chronic disease and medication as a significant barrier. The lack of time to develop individualized transition protocols was reported by 43 (61.41%) respondents, and 46 (65.7%) highlighted the absence of a transition expert at each center.

A PCA regarding barriers as active observations and medical specialty as active variables was performed, and the two principal components covered 91.19% of the variability (F1: 76.12% and F2: 15.08%). Pediatric specialists identified the adolescents’ lack of knowledge regarding the chronic disease, the followed medication, the possible evolution in the absence of medication, the lack of time to create an individualized transition protocol, and the shortage of doctors in the primary care network to assist in the transition process. The psycho-emotional attachment of the patient and the family to the pediatric team was highlighted by the AG and PCP (Figure 2).

Regarding training, 66 (94.3%) respondents supported introducing a chapter on transitional care in the training curriculum for pediatric and primary care specialties.

## Discussion

Data included in this paper represent the first description of the transition practices of doctors caring for adolescents with chronic digestive diseases from the Northeastern region of Romania.

We identified a lack of transition protocols recognized by 63 (90%) specialists. The high proportion of 68 (97.1%) respondents recognizing the need for a multidisciplinary team underscores the complexity of transition care and the necessity for collaborative approaches involving various healthcare professionals. The identification of the lack of a transition specialist as a critical barrier by a substantial majority of respondents (n = 46; 65.7%) indicates a clear gap in expertise and coordination within the current system. Transition specialists can provide dedicated oversight and ensure that all aspects of the patient’s care are managed effectively during the transition period, which is crucial for maintaining continuity and quality of care.

We report important data on translational practices: 35 (50%) doctors transitioning their patients based on the last medical letter and 26 (38.2%) by simply discussing with patients and their parents. These practices highlight a need for more robust and detailed transition protocols reinforced by written content to ensure all necessary information is conveyed accurately and completely.

Only one participant declared including a discussion with an adult-oriented specialist; on the other hand, 19 (31.7%) doctors declared being contacted by patients or families for medical emergencies. We assume that this high number of patients in need of assistance is due to the identified transition malpractices and the fact that, according to recent literature, after reaching adulthood, young patients may feel ‘lost in the system’ and may have the tendency to reach out to the pediatric specialist in cases of emergencies [7,8].

While the benefits of joint adult and pediatric consultations are established, the optimal frequency and number of consultations remain unclear. Various models suggest biannual mixed consultations starting at the age of 16 until transfer at 18, or quarterly multidisciplinary consultations from the ages of 16-18, followed by a final consultation with an adult physician [9-11].

Cole et al. recommend initiating consultations with both pediatric and adult gastroenterologists at 15 years of age, with transfer occurring at 16 years [12]. Canadian authors note that some doctors continue to support patients up to the age of 18-19 or until high school graduation, with transition initiation varying from one or two months to several years before transfer [13].

The National Institute for Health and Care Excellence suggests starting transition plans at the age of 13-14, while the North American Society for Pediatric Gastroenterology, Hepatology, and Nutrition recommends transitioning inflammatory bowel disease (IBD) patients by the age of 18 [14, 15]. The American Academy of Pediatrics, the American Academy of Family Physicians, and the American College of Physicians recommend individualized transfer to adult care between the ages of 18 and 21 [16,17]. 

We identified significant variations in physicians' perceptions regarding the optimal timing for transitioning patients with chronic conditions from pediatric to adult care. Both PCP and GP generally recommend initiating the transition at younger ages, while both pediatric and adult gastroenterologists prefer starting the process at older ages (p<0.05).

Regarding the actual transfer, PCP and AG advocate for earlier transfer ages (p<0.05), likely due to their experience with adult patients and the aim to ensure stable disease management and avoid risks associated with premature transitioning. Adult specialists seem to encourage patient independence and promote self-sufficiency skills to help adolescents navigate emerging challenges, which is crucial for those with chronic conditions. However, while evidence supports fostering self-management skills, these models are not typically developed for younger patients and are not routinely integrated into pediatric care [18]. 

Best practices for transition include promoting medical independence, early preparation for the transition, and enhancing communication among patients, families, and both pediatric and adult providers [19].

The Italian Societies of Gastroenterology indicate that prolonged interactions with pediatric medical staff can create an unbalanced relationship among patients, families, and the medical team due to a sense of exclusivity in care [20]. Similarly, in our study, 52.84% of respondents identified the psycho-emotional attachment of patients and families as a significant barrier to the transition process, potentially hindering communication with new providers and affecting care quality. To mitigate this, a multi-stage transition protocol involving collaboration between pediatric and adult medical teams is recommended. Joint consultations and gradual introductions to the new team can ease emotional strain. Additionally, providing psychological support for patients and families throughout the transition is essential to addressing emotional imbalances.

In our research, 24 (34.3%) of responders declared not encouraging teenagers to independently manage their chronic condition. The importance of self-management is related to the improvement of healthcare behavior, decrease in hospital admissions, and, consequently, improvement of patients’s quality of life [21]. We assume that the findings in our research are related to fear of self-medication and interactions between medications for chronic conditions and other possible self-administered drugs for acute intercurrent events, especially regarding antibiotics and antibiotic resistance.

An important consideration during the transition is the management of antibiotic resistance, particularly in the context of *Helicobacter pylori *(*H. pylori*) infection. The rise of antibiotic resistance poses a significant challenge in treating *H. pylori*, leading to treatment failures and disease recurrence [22]. Pediatric patients are vulnerable to antibiotic-resistant infections due to factors such as frequent antibiotic exposure and immature immune systems [23]. Transitioning pediatric patients to adult care requires a focus on combating antibiotic resistance for optimal treatment results. This period offers a pivotal opportunity to introduce antimicrobial stewardship initiatives, aiming to minimize unnecessary antibiotic usage, promote prescribing practices, and educate patients about antibiotic resistance. By incorporating strategies to address antibiotic resistance into the transition process, healthcare providers can safeguard patients' long-term health outcomes and foster responsible antibiotic use in adult care settings.

There is a growing interest in utilizing digital and online tools to facilitate the transition period in healthcare. These tools have the potential to enhance communication between patients and healthcare teams, particularly given the widespread access to social media and online communication applications. However, despite this increasing interest, the specialized literature indicates a shortage of both physical and electronic resources [13]. 

The statistical analysis conducted revealed that both GPs and PGs demonstrated a strong correlation (0.836; p<0.05) with the belief that a higher number of resources is more useful in the transition process, compared to other specialties. These findings support advocacy efforts for comprehensive care models that integrate various resources and services into the transition process.

Initial steps towards a national electronic patient’s medical history have been made in Romania, where the IBDPROSPECT project offered a new perspective regarding doctors’ online access to the medical information of adult IBD patients. The project aimed at creating an electronic infrastructure for data collection on patients with IBD at a national level, thus facilitating the monitorization of patients’ evolutions in a continuous, prospective manner. This registry includes variables such as epidemiological data, personal and family medical history, blood test results, endoscopic or histological data, and former and current therapies [24].

Researchers have proposed and developed another project to facilitate data access for chronic patients, a pilot electronic model of a children's chronic disease register adapted to the Romanian healthcare system and to standard requirements regarding data protection [25]. However, the study was discontinued, and up to this moment, in Romania, there is no national register for pediatric-onset chronic digestive disease patients and no electronic devices to offer access to information regarding these patients to doctors or patients.

Authors investigating populations with chronic diseases propose the use of artificial intelligence (AI)-powered communication tools, which can provide personalized, on-demand support to adolescents on the verge of transition, helping them navigate the process and offering them access to real-time education and information. However, constant and accurate updates and expert supervision of such tools are mandatory in order to ensure the high quality of generated data [26].

Addressing these barriers requires time and governmental support, which involves the allocation of financial and human resources to enable translational policies. Although resources such as support groups and informational brochures can be provided at a clinic or hospital level, the implementation of other resources, such as online platforms, demands a multi-institutional approach. Enhancing adolescents' understanding of their chronic disease could be accomplished through the development of individualized, multi-stage transition protocols where a transition coordinator, the patient, and their family would collaborate to achieve specific objectives. 

Our study presents several limitations that should be mentioned. First, the number of responders was limited to 70 participating doctors. Secondly, there could be potential social desirability bias concerns regarding data extracted from self-reported questionnaires; however, participants were reassured that their answers would be transmitted anonymously, which helps minimize the potential bias. Thirdly, this study does not include data on patients and their families’ perspectives. This study is part of a larger research on transition strategies with the final aim of creating the first protocol for the transition of Romanian teenagers suffering from chronic digestive diseases. We recommend assessing the transition readiness of adolescents and considering the concerns, anxieties, and hopes of their families, as these factors can provide valuable insights for improving support during the transition process.

## Conclusions

This study highlights the significant barriers and resources identified by healthcare specialists in the transition of adolescents with chronic digestive diseases. By addressing identified barriers and implementing recommended resources, we can ensure smoother transitions with the ultimate goal of fostering independence in young patients in order to empower them to manage their health effectively as they reach adulthood. As we look to the future, the path forward lies in building robust, patient-centered transition frameworks that seamlessly integrate pediatric and adult healthcare systems; the journey ahead requires collaboration, innovation, and a commitment to continuous improvement in translational care.
